# Efficacy and safety of artemisinin-based combination therapies for the treatment of uncomplicated malaria in pediatrics: a systematic review and meta-analysis

**DOI:** 10.1186/s12879-021-06018-6

**Published:** 2021-04-07

**Authors:** Workineh Shibeshi, Getachew Alemkere, Assefa Mulu, Ephrem Engidawork

**Affiliations:** Department of Pharmacology and Clinical Pharmacy, College of Health Sciences, Addis Ababa University, Addis Ababa, Ethiopia

**Keywords:** Efficacy, Safety, Artemisinin-based combination, Systematic review, Meta-analysis

## Abstract

**Background:**

Malaria is a major cause of morbidity and mortality in pediatrics in malaria endemic areas. Artemisinin-based combination therapies (ACTs) are the drugs of choice for malaria management particularly across malaria-endemic countries. This systematic review and meta-analysis was performed to assess efficacy and safety of ACTs for uncomplicated malaria in pediatric populations.

**Methods:**

A body of evidence was searched for published ACT trials until March 06, 2020. The search was focused on efficacy and safety studies of ACTs for uncomplicated malaria in pediatrics. PubMed library was searched using best adapted search terms after multiple trials. References were exported to the endnote library and then to Covidence for screening. Data was extracted using the Covidence platform. The per-protocol analysis report for the efficacy and the intention-to-treat analysis for the safety were synthesized. Met-analysis was carried using Open Meta-Analyst software. Random effects model was applied and the heterogeneity of studies was evaluated using I^2^ statistic.

**Results:**

Nineteen studies were included in the final analysis. Overall, crude, PCR-corrected *P. falciparum* malaria treatment success rate was 96.3 and 93.9% for day 28 and 42, respectively. In the subgroup analysis, PCR-corrected adequate clinical and parasitological response (ACPR) of dihydroartemisinin-piperaquine (DP) was 99.6% (95% CI: 99.1 to 100%, I^2^ = 0%; 4 studies) at day 28 and 99.6% (95% CI of 99 to 100%, I^2^ = 0%; 3 studies) at day 42. Nine studies reported ACT related adverse drug reactions (ADR) (8.3%, 356/4304). The reported drug related adverse reactions ranged from 1.8% in DP (two studies) to 23.3% in artesunate-pyronaridine (AP). Gastrointestinal symptoms were the most common ACT related adverse effects, and all ADRs were reported to resolve spontaneously.

**Conclusion:**

ACTs demonstrated a high crude efficacy and tolerability against *P. falciparum*. The high treatment success and tolerability with low heterogeneity conferred by DP has implication for policy makers who plan the use of ACTs for uncomplicated falciparum malaria treatment in pediatrics.

**Supplementary Information:**

The online version contains supplementary material available at 10.1186/s12879-021-06018-6.

## Background

Despite decades of experience while practicing control measures, malaria is still a major public health challenge, with 219 million new cases and 435,000 deaths globally. Sixty one percent (266, 000) of the death are being among under 5 years old children. The World Health Organization (WHO) African region accounted for 92 and 93% of the malaria cases and deaths, respectively [1]. Despite being home for malaria, the WHO African region accounted for 88% of the 172,000 fewer global death reports in 2017 as compared to 2010 [1].

One of the key strategies devised in the advent of malaria management was emphasizing on the importance of early diagnosis and treatment [2]. However, this was progressively disadvantaged by emergence of resistance of malaria parasites to the existing treatment options. Particularly, a global resistance of *P. falciparum* to chloroquine and the sulphadoxine-pyrimethamine prompted the 2001 WHO expert panel to suggest use of artemisinin-based combination therapy (ACT) for uncomplicated *P. falciparum* malaria management [3]. WHO recommends ACTs as the first-line and second-line treatments for uncomplicated *P. falciparum* malaria as well as for *P. vivax* malaria resistant to chloroquine.

ACTs integrate an artemisinin derivative with a non-artemisinin partner drug. Although the efficacy of ACT is dependent on both agents, the artemisinin is critical to reduce the parasite biomass during the first 3 days of treatment. The partner drug then helps to eliminate (cure) the remaining parasites [4]. Hence, the two agents work together to attain effective clinical and parasitological cures and believed to protect each other from development of resistance [3]. ACTs are available either as fixed-combination products co-formulated in the same tablets or capsules, or loose preparations co-administered in separate tablets or capsules.

Although there is a wide range of treatment failure reports for the ACTs, they are still mainstay drugs for averting uncomplicated malaria from progressing to severe disease and death [5–12]. To preserve therapeutic efficacy of ACTs, WHO recommends malaria-endemic countries to perform routine antimalarial drug efficacy monitoring at sentinel sites at least once every 24 months. This recommendation is particularly directed at determining the day 28 or 42 proportion of treatment failures. If the treatment failure is 10% or more, a change in the national treatment policy is recommended. National malaria programs are also recommended to adopt medicines with a pharmacologic cure rate of greater than 95% [4].

Currently, maintaining the efficacy of ACTs for the management of malaria is a global health priority [1]. Therapeutic efficacy studies conducted between 2010 and 2017 showed that ACTs have greater than 95% efficacy outside the Greater Mekong subregion (GMS). Luckily, no artemisinin (partial) resistance has been reported from Africa in this document [1].

ACTs are generally tolerable drugs [5, 8, 13]. One old review showed high tolerability of artemisinin drugs over other antimalarial drugs, particularly quinines [14]. Dose-dependent neurotoxicities, cardiovascular toxicities and gastrointestinal side effects were reported for artemisinin use in animal and human studies [14, 15]. Artemisinins can have a cumulative toxicity if used for a prolonged period and at high dose than recommended, probably due to unknown long-living metabolites [14, 15].

Being safe and effective, several ACTs have been widely recommended for the management of uncomplicated malaria [16] and had significantly decreased the morbidity and mortality of malaria in pediatrics [17]. From 19 household surveys in sub-Saharan Africa conducted between 2015 and 2017, 29% (Interquartile range: 15–48%) children aged under 5 years had received any antimalarial drug. They were more likely to receive ACTs if they had sought care in the public than the private sector [1] Although ACTs are widely used for the treatment of malaria in pediatrics, there are limited information about the efficacy and safety as well as the dosage of ACTs in young infants due to the marked difference in the metabolic characteristics of this group of the population [18]. Manual conversion of the formulations that may result in under-dosing for this group of population is also one area that derived the development of pediatric formulations. One systematic review comparing the pediatric and standard dosage formulations among this population showed a high efficacy and overall high tolerability of ACTs [8].

To date, the success of ACTs in the management of uncompleted malaria in pediatrics have been threatened by resistance. The spread of resistance to the areas with the highest malaria burden areas like the sub-Saharan Africa region would be a major disaster. This requires containing resistance with all available means. We believe, this study is one such effort in the process of preserving the efficacy of ACTs.

To the best of our knowledge, there was no comprehensive systematic review and meta-analysis study that address the efficacy and safety of ACTs in the pediatric population. Therefore, this systematic review and meta-analysis was aimed at exploring and synthesizing the existing body of evidence on the efficacy and safety of ACTs among pediatrics. The primary endpoint considered was polymerase chain reaction (PCR)-corrected day 28 adequate clinical and parasitological responses (ACPR), while day 42 ACPR and safety were considered as secondary endpoints.

## Methods

### Search strategies

Studies included in this systematic review and meta-analysis were screened using the preferred reporting items for systematic review and meta-analysis (PRISMA) statement. The following systematic search strategy was applied to search the PubMed database: artemisinins [tiab] OR artemis*[tiab] OR artesun*[tiab] OR artemotil [tiab] OR arteether [tiab] OR dihydroarte*[tiab] OR qinghaosu [tiab] OR qinghaosu [tiab] OR qinghaosu [tiab] OR arteflene*[tiab] OR artemether*[tiab]) AND (“combination therapy”[tw] OR ACT [tw]) AND (treatment outcome [MeSH Terms]) AND (uncomplicated malaria) AND (vivax [tiab] OR falciparum [tiab] OR non-mixed species [tiab]) AND (child* OR infant* OR adolescen* OR pediatric*. Based on these search terms, 153 published studies were retrieved by March 06, 2020. After applying the following filtration criteria: English, human, pediatrics (of all categories available), and full text; 138 papers were retained. Based on a random title skimming for terms of exclusion, 125 papers were clipped from PubMed and exported to endnote library.

**Fig. 1:**
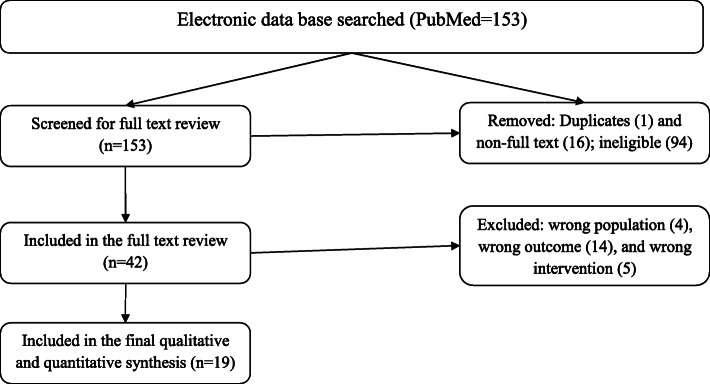
PRISMA chart for study selection

Sixteen papers that do not have a full text PDF for off-line work and 5 reviews were removed. Further, endnote smart group analysis was applied to exclude 22 unwanted studies due to the study population mismatch. The sub-group analysis was applied to group the studies usually based on the key words (as indicated in the exclusion criteria such as a word adult) present in the titles and/or abstracts of the respective papers. Then they will be skimmed and removed if they do not match the inclusion criteria. From the remaining 82 studies 40 were removed based on the inclusion criteria. The remaining 42 articles were exported to covidence for further screening and data extraction. Among the 42 articles reviewed for full text, the outcome of interest was not addressed in 14 studies. Two studies involving adults, 5 different non-ACT interventions and 2 mixed infections were excluded, leaving 19 studies for final data extraction (Fig**.** 1).

### Eligibility criteria

Original articles that examined ACT for the treatment of uncomplicated *P. falciparum* or *P. vivax* malaria were considered in this systematic review and meta-analysis. PICOS format was applied (Table 1). The primary outcome of this review was the efficacy of ACT reported as ACPR at day 28 with a PCR correction. The secondary outcome measures were PCR- corrected ACPR at day 42 and the frequency of adverse drug reactions (ADR). ADR was defined as ‘signs and symptoms or abnormal laboratory value reported as drug related adverse events by the author/s’. Studies that tested non-artemisinin and artemisinin monotherapy and those assessing treatment outcomes at days less than 28 were excluded from this systematic review and meta-analysis.

**Table 1:** Inclusion criteria based on the PICOS format for the study conducted

PICOS	Inclusion Criteria	Exclusion Criteria
Participants	● Pediatrics including neonateswith microscopically confirmed, uncomplicated *P. falciparum* orVivax malaria. ● With mono-infections	● In-vitro studies ● Adults, ● All with sever malaria ● All with other types of malaria and co-infections/mixed infections
Interventions	● Treatment at least three-day course of an ACT (fixed dosed, co-blistered, or individually packaged (loose))	● Prophylaxis ● Studies that do not report ACT ● For non-comparative trials, those with ACT and other drugs with antimalarial properties ● Artemisinin mono-therapy
Comparison	An ACT with or without comparative arm interventions (ACT with ACT, ACT with non-ACT, ACT alone)	● Studies that do not report an ACT
Outcome measures	● Efficacy: PCR corrected day 28 and 42 ACPR and ● Safety: Adverse drug events including associated deaths	● Follow-ups less than day 28 ● Retreatments
Studies	● All study designs globally ● Published in English ● No restriction to number of authors ● Published until March 06, 2020 ● phase III/IV clinical trials	● News, communication, qualitative studies, case reports ● all non-published studies and published in non –English languages ● Phase I/II trials.
Overall	● Must fulfill all above inclusion criteria’s	● Must not include either of the above

### Selection of studies

One reviewer assessed each study for inclusion in this review using endnote and covidence based on a predefined inclusion criterion. For studies that were ineligible, the respective reasons for their exclusion were reported according to the PRISMA algorithm (Fig. 1).

### Data extraction and management

The reviewer extracted the data two times using different user names in a Covidence (non-Cochrane) data extraction template. The data were extracted for the following study characteristics: first author, year of publication, study setting, study design, baseline characteristics of trial participants, malaria species, and antimalarial drug tested, dose, route, duration and drug ratios of the combinations. Data regarding treatment outcome measures including efficacy (ACPR treatment success on days 28 and 42) and ADRs were extracted and included in the systematic review and meta-analysis. The collected data, particularly the outcome (day 28 and 42, and ADRs), were rechecked several times. In addition, a second reviewer also cross-checked all the data entries and the abstracted figures. The data for total number of randomized and analysed, loss to follow-ups and/or exclusions from the analysis, reinfections, and recrudesces were captured or calculated for the two follow-up days for each treatment groups. The per-protocol analysis and when available the intention-to-treat analysis and Kaplar-Meier analysis were documented. However, due to inconsistencies in the latter two reports, the PCR corrected per-protocol analysis was analysed and reported. In case of missing values for the day 28 or 42 number of events, we tried to calculate it from the percentage ACPR reports using all the available information as indicated above. The medication adverse effect reports were collected with a particular attention to the authors comment on the drug-event relationship. We documented the number of participants experiencing medication related events and the total number of randomized participants.

### Risk of bias assessment

We used the Cochrane risk of bias 2 (RoB 2) assessment excel tool to explore sources of bias in included randomized trials. This scale evaluates biases arising from the following five domains: the randomization process, deviations from intended interventions, missing outcome data, measurement of the outcome, and selection of the reported result. Risk of bias was categorised as high, low or some concerns. If any domain was judged as high risk, then the trial had labelled high risk of bias. Single-arm trials were not assessed further as they already have a high risk of bias by their nature.

### Data synthesis

ACPR was utilized as an indicator for efficacy assessment. ACPR was defined by WHO as lack of parasitemia to the treatment by the end of day 28 or 42 irrespective of axillary temperature in patients that do not meet any of the criteria for early treatment failure, late clinical failure or late parasitological failure [19–21]. Efficacy outcomes of the included studies were evaluated at the 28th and 42^nd^day of treatment. All outcomes of the included studies were defined based on PCR genotyping. The per-protocol analysis and the intention-to-treat analysis were used for efficacy and safety assessment, respectively.

### Data analysis and heterogeneity assessment

OpenMeta-Analyst software for Windows [http://www.cebm.brown.edu/openmeta/#] was used for the meta-analyses. The I^2^ statistic was used to assess heterogeneity of the included studies. Heterogeneity was conventionally defined with I^2^ > 50 [22]. Based on this, the included studies were highly heterogeneous (day 28 efficacy I^2^ = 85.9%; 19 studies). The random effects model was used to combine the included studies. A sub-group analysis was carried out for different ACTs, year of publication and study design. The artesunate-amodiaquine (ASAQ) arms were also sub-grouped based on drug formulations (fixed versus loose). Drug related adverse effects were computed and compared for the different ACTs regimens.

## Results

### Study characteristics

A systematic strategy was used to search 153 articles from PubMed (Fig. 1). Among the 42 full text studies, 19 relevant research topics were identified through mining of the available literature up to 6 March 2020 [23–41]. The study characteristics are indicated in Additional file 1. All finally included studies were from Africa and conducted between 2007 to 2019. Among 19 studies, 9 were randomized controlled trials, RCTs [24, 29–32, 34, 36, 38, 40], of which 2 were double blind [30, 36], 2 single blind [31, 38] and 5 open label trials [24, 29, 32, 34, 40]. The remaining 10 studies were single arm or non-comparative studies with no clear randomization [23, 25–27, 33–35, 37, 39, 41]. By the type of malaria infection, all selected studies were conducted on *P. falciparum*.

In 19 studies, 9121 (range: 14–914) participants were initially enrolled. Of them, 8194 (range: 11–765) and 7932 (range: 11–765) participants were included in the PCR uncorrected and PCR-corrected per-protocol analysis of day 28 efficacy, respectively. The studies with the smallest (*n* = 15) and largest (*n* = 914) sample size for the treatment groups were that of Ramharter et al., 2008 [37] and Premji et al., 2009 [36], respectively.

The trials examined a total of 40 treatment groups, of which 14 groups (14 studies) received artemether-lumefantrine (AL), 15 groups (14 studies) artesunate-amodiaquine (ASAQ), 4 groups (4 studies) Dihydroartemisinin–Piperaquine (DP), 3 groups (2 studies) other ACTs and 4 groups (1 study) received non-ACTs (non-ACTs were excluded from the meta-analysis) (Table 1). All the ACTs were given orally for 3 days. Dosing schedules for all ACTs were based on standard recommendations, except one dose-escalation study aimed at assessing safety of pyronaridine-artesunate (AP) [37]. This study used the 6: 2 mg/kg tablet, 9: 3 mg/kg tablet, 12: 4 mg/kg tablet and 9: 3 mg/kg granules. The data were extracted for all but analyzed only for 9: 3 mg/kg tablet and granules.

The baseline characteristics were comparable in 12 studies [24, 28–31, 33, 34, 36–38, 40, 41] and different in at least one parameter for the remaining 7 studies [23, 25–27, 32, 35, 39]. Different inclusion criteria were applied for 2 studies [25, 35]. The study by Shayo et al., (2014) assessed coartem among under five and above 5 years of children and there was a difference in sex distribution among the two age groups [39]. There was a significant difference in terms of sex, weight and other characteristics for 2 studies [26, 27]. The significant difference was limited to the geometric mean difference in one study [32]. Seven studies included pediatrics between 6 months to 59 months [23–26, 30, 37, 38]. Two multicenter (six site) studies included different age categories (6 to 59 months in four and 6 months to 12 years in two sites of each) in different treatment centers [22, 32]. The other studies included under nine [20], 10 [29, 35, 36], 12 [28, 31], 13 [21], 14 [34] and 15 years children [27, 33]. Nine studies had participant retention rates > 90% [25, 28, 29, 32–35, 37, 39, 40] and no study had lost to follow- up of 20% and above.

In all studies, patients were followed up to day 28. However, only five studies had a follow-up to day 42 [25, 35–38]. For two studies, the follow-up to day 42 was made only for the DP arm [25, 35].

Except in one study that used the modified criteria [24], the WHO clinical and parasitological criteria were used to assess treatment outcomes. Hence, this study utilized the PCR corrected ACPR on day 28 and day 42 based on the WHO recommendation to assess the efficacy.

### Efficacy assessment

Two treatment groups in the artemether-lumefantrine (AL) arm in two studies conducted in Angola [35] and Ghana [29] showed greater than 10% (in the range of 10 to 15%) treatment failure on day 28. Five ASAQ treatment groups showed PCR corrected day 28 treatment failure of more than 5% (within the range of 5 to 10%) in Angola [25], Burkina Faso [40], Kenya [41], Madagascar [30], Ghana [29] and Tanzania [28]. One multi-country study (Burkina Faso, Ghana, Kenya, Nigeria, Tanzania) on CDA and AL showed more than 5% (in the range of 5 to 10%) treatment failure both in the day 28 and day 42 [36]. All the remaining ACT treatment groups had a treatment failure of less than 5% at day 28 and day 42.

### Day 28 efficacy assessment

Seventeen treatment groups (in 9 studies) showed 100% success rate (Table 2).

**Table 2:** Summary characteristics of studies with 100% efficacy of artemisinin combination therapy at day 28 among P. falciparum patients

Author, publication year	Country	Setting	Drug	Age	n	N	p
Davlantes 2018 [25]	Angola	Benguela (stable mesoendemic transmission)	DP	< 12 years	85	85	100
			ASAQ	< 12 years	90	90	100
		LundaSul-hyperendemic transmission)	DP	< 5 years	89	89	100
Ojurongbe 2013 [34]	Nigeria	University hospital, transmission throughout the year	AL	6 m to 12 years	89	89	100
			ASAQ		71	71	100
Plucinski 2017 [35]	Angola	Benguela (stable mesoendemic transmission)	ASAQ	6 m to 12 years	66	66	100
		LundaSul-hyperendemic transmission)	DP		76	76	100
			ASAQ		56	56	100
Ramharter 2008 [37]	Gabon	Hospital. transmitted perennially	AP (tablet)	2–14 years	13	13	100
			AP (Granule)	2–14 years	14	14	100
Sawa 2013 [38]	Kenya	moderate transmission intensity	DP	6 m to 10 years	137	137	100
Shayo 2014 [39]	Tanzania	Health Center. Moderate to high transmission	DP	6 months to 10 years	40	40	100
			AL	6 m to 10 years	21	21	100
Mens 2008 [31]	Kenya	primary health centers, transmission low and predominantly seasonal	DP	6 m to 12 years	67	67	100
			AL	6 m to 12 years	66	66	100
Kabanywanyi 2007 [28]	Tanzania	health facilities, perennial with seasonal peaks	AL	6 to 59 months	86	86	100
Dorkenoo 2012 [27]	Togo	Urban, university and childs hospital, endemic or seasonal transmission	AL	6 to 59 months	538	538	100

**Fig. 2:**
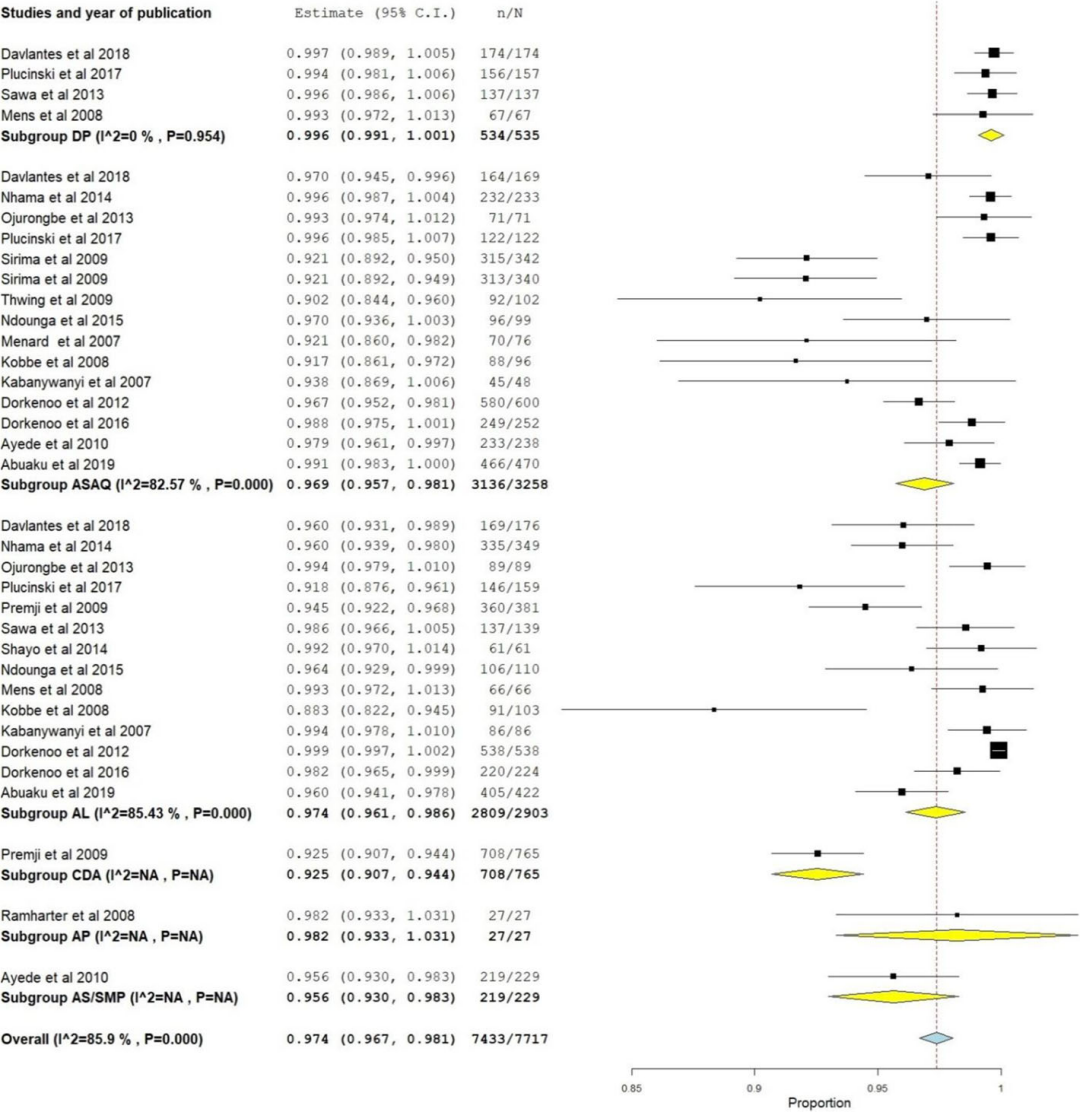
Meta-analysis of day 28 efficacy of artemisinin combination therapy administration (PR: DerSimonian-larid random-effects untransformed proportion).Abbreviations:- C.I.: confidence interval, n: events, N: total in the per-protocol analysis, DP: dihydroartemisinin-piperaquine, ASAQ: Artesunate–Amodiaquine, AL: Artemether–Lumefantrine, CDA: Chlorproguanil-dapsone-artesunate, AP: artesunate-pyronaridine, ASSMP: Artesunate-Sulphamethoxypyrazine-Pyrimethamine

Overall, day 28 PCR corrected malaria treatment cure rate was 96.3%. Treatment with DP (99.8%) was found to have the higher cure rates than AL (96.8%) and artesunate-amodiaquine (ASAQ) (96.3%) (Table 3). This high cure rate was, however, highly affected by heterogeneity as discussed below (Fig. 2).

**Table 3:** Day 28 treatment success rate for artemisinin combination therapies among P. falciparum infected patients

No.	Drug used	Number of studies (treatment groups)	Drug efficacy (PCR- corrected day 28)
1	Artemether–Lumefantrine - (AL)	14 (16)	2809/2903 (96.8%)
2	Artesunate–Amodiaquine - (ASAQ)	14 (17)	3136/3258 (96.3%)
3	Dihydroartemisinin–Piperaquine - (DP)	4 (6)	534/535 (99.8%)
4	Artesunate-Sulphamethoxypyrazine-Pyrimethamine - (AS+SMP)	1 (1)	219/229 (95.6%)
5	Pyronaridine:artesunate (PA) (tab & granule)	1 (2)	27/27 (100%)
6	Chlorproguanil-dapsone-artesunate (CDA)	1 (1)	708/765 (92.5%)
7	Total		7433/7717 (96.3%)

We tried to perform meta-analysis for day 28 efficacy. However, we found high heterogeneity (overall I^2^ = 85.9%; 19 studies, 36 treatment groups) (Fig. 2). Subgroup analysis was carried out to assess the heterogeneity. In the subgroup analysis, DP was found to have an efficacy of 99.6% with 95% CI of 99.1 to 100% (I^2^ = 0%; 4 studies). A significant heterogeneity precluded utilization of the subgroup effect size estimates for AL (I^2^ = 85.43%; 14 studies) and ASAQ (I^2^ = 82.57%; 14 studies, 15 treatment groups). Further, we also tried to subgroup the ASAQ data into loose and fixed dose formulation, but results were not reported due to a significant heterogeneity. Likewise, though not successful, subgroup analyses were also carried out by study designs (RCT versus non-RCT) and year of publication.

### Day 42 efficacy assessment

**Fig. 3:**
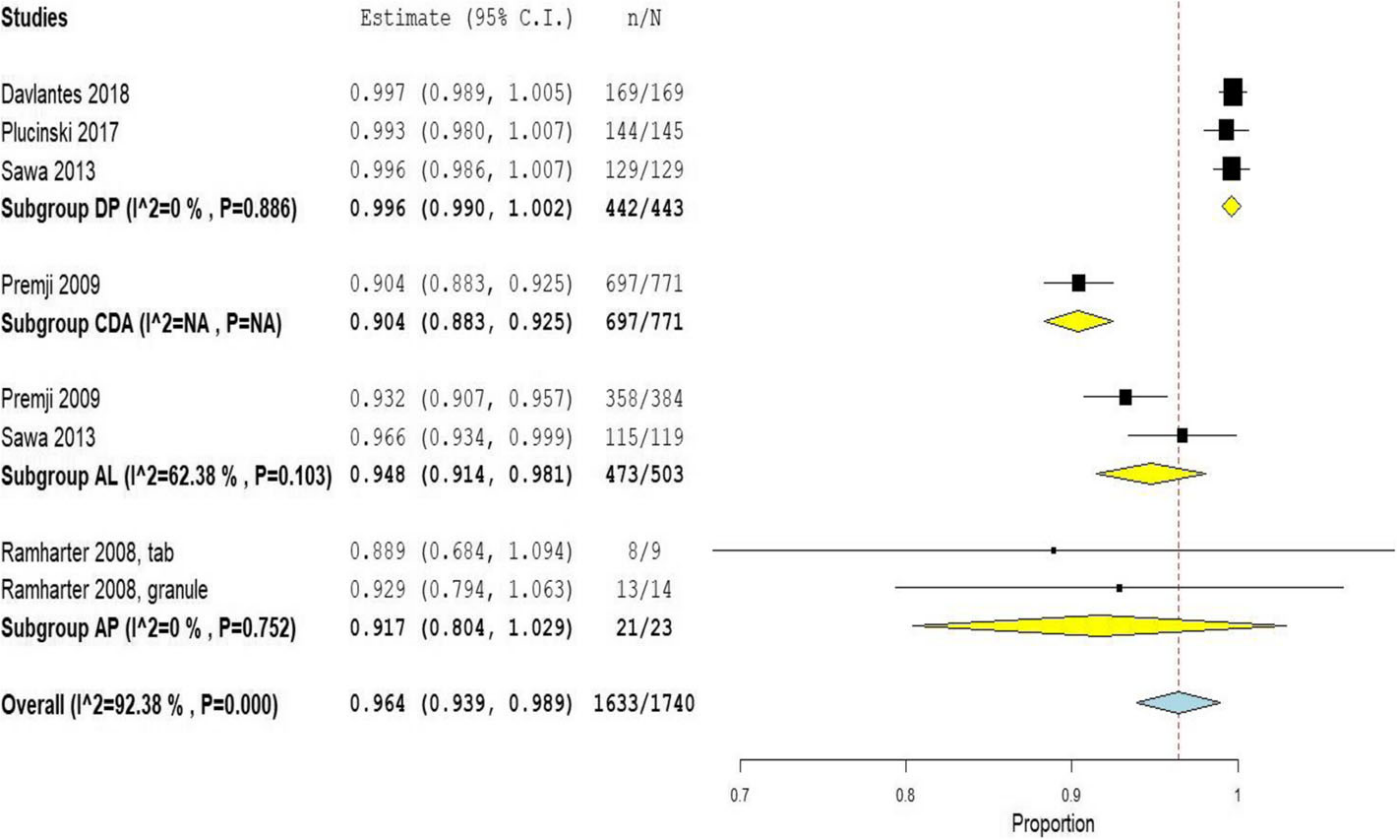
Meta-analysis of day 42 efficacy of artemisinin combination therapy administration (PR: DerSimonian-larid random-effects untransformed proportion).Abbreviations:- C.I.: confidence interval, n: events, N: total in the per-protocol analysis, DP: dihydroartemisinin-piperaquine, CDA: Chlorproguanil-dapsone-artesunate, AL: artemether-lumefantrine, AP: artesunate-pyronaridine

Five studies presented a complete PCR corrected ACPR data for day 42. DP was assessed in three studies, AL in two studies, CDA in one study and AP (in different doses) in one study. Two studies were RCT [36, 38] and the remaining three were non-RCT [25, 35, 37] (Table 4). We also performed meta-analysis for day 42 efficacy and found high heterogeneity (overall I^2^ = 92.4%; 5 studies, 8 treatment groups). In the subgroup analysis, DP was found to have an efficacy of 99.6% with 95% CI of 99 to 100% (I^2^ = 0%; 3 studies) (Fig. 3).

**Table 4:** Day 42 treatment success rate of artemisinin combination therapies among P. falciparum patients

Author	Country	Design	Age	Drugs	Day 42 ACPR (n/N, %)
Davlantes 2018 [25]	Angola	Non-RCT	< 12 years	DP	169/169, 100%
Plucinski 2017 [35]	Angola	Non-RCT	6 m to 12 years	DP	144/145, 99.3%
Premji 2009 [36]	Burkina Faso, Ghana, Kenya, Nigeria, Tanzania	RCT, phase III trial	1 to 15 years	CDA	697/771, 90.4%
				AL	358/384, 93.2%
Ramharter 2008 [37]	Gabon	Non-RCT	2–14 years	AP 9:3-mg/kg tabs	8/9, 88.9%
				AP 9:3-mg/kg granule	13/14, 92.9%
Sawa 2013 [38]	Kenya	RCT	6 months to 10 years	AL	115/119, 96.6%
				DP	129/129, 100%
**Total**					**1633/1740, 93.9%**

### Safety assessment

Except for one study [38], all 18 studies (94.7%) reported adverse events. While 9 studies did [24, 25, 31, 33, 36, 37, 39–41], seven studies [23, 26–28, 30, 32, 35] did not report correlation for drug and adverse events. The remaining two studies [29, 34] provided vague information about drug and adverse event association. All but one study [36] claimed the absence of severe adverse effects or ruled out severe adverse effects as drug related [29]. Though Premiji et al., [36] reported mild adverse effects in 184 (20%) patients in the CDA group and 86 (19%) in the AL group as probably or possibly drug-related, they failed to demonstrate the association of the adverse effects with the administered drugs. However, they suggested that this high percentage of adverse effects, particularly for CDA, could probably be attributed to oxidative hemolysis, secondary to G6PD-deficiency in patients receiving the treatments.

Adverse effects for the 15 studies were reported based on the intention-to-treat analysis. Three studies [24, 25, 39], however, reported based on the per-protocol analysis. Despite this, we used the intention to treat analysis to calculate the pooled estimate for the nine studies that reported drug related adverse effects. There was no severe ADRs or deaths in all the 9 included studies.

Among those who commented on the association to ACT, adverse medication effects were observed in 356/4304 (8.3%) of the patients [24, 25, 31, 33, 34, 36, 37, 39–41]. After removing three studies [24, 34, 36, 37] that had constraints in distinguishing specific drug- related adverse effects, the most common mild adverse drug effects reported by the remaining 6 studies [25, 31, 33, 39–41] were (vomiting (*n* = 22, in 4 studies), diarrhea (*n* = 6, one study), weakness (*n* = 4, one study), sweating (*n* = 3, one study), and nausea (n = 3, one study), which were resolved spontaneously. The reported drug related ADRs ranged from 1.8% (for DP) to 23.3% (of 24-h regimen of AP) (Table 5).

**Table 5:** Adverse drug reactions of artemisinin combination therapies among P. falciparum patients (9 studies included)

No.	Drug used in the study	Number of studies mentioned ADRs as drug related	Frequency (%) of patients with ADRs relevant to specific treatment regimen
1	Artemether–Lumefantrine (AL)	5	88/1263 (6.9%)
2	Artesunate–Amodiaquine (AS+AQ)	5	69/1574 (4.4%)
3	Dihydroartemisinin–Piperaquine (DP)	2	5/273 (1.8%)
4	Artesunate-Sulphamethoxypyrazine-Pyrimethamine (AS+SMP)	1	3/250 (1.2%)
5	Pyronaridine:artesunate (PA) (tab & granule)	1	7/30 (23.3%)
6	Chlorproguanil-dapsone-artesunate (CDA)	1	184/914 (20.1%)
	Total		356/4304 (8.3%)

**Fig. 4:**
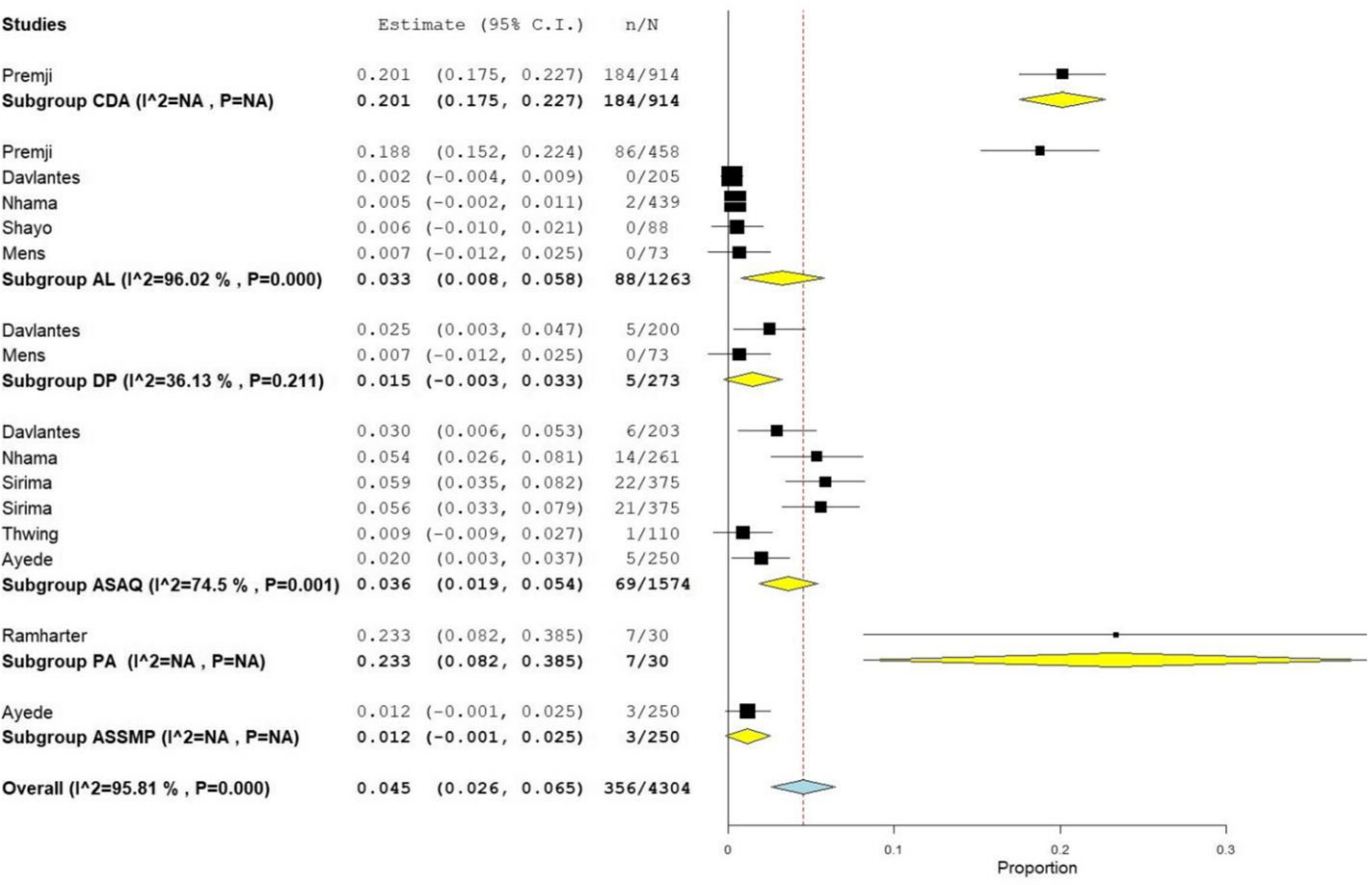
Meta-analysis of safety of artemisinin combination therapy administration (PR: DerSimonian-larid random-effects untransformed proportion). Abbreviations: - C.I.: confidence interval, n: events, N: total in the intention-to-treat analysis, CDA: Chlorproguanil-dapsone-artesunate, AL: Artemether–Lumefantrine, DP: dihydroartemisinin-piperaquine, ASAQ: Artesunate–Amodiaquine, PA: pyronaridine-artesunate, ASSMP: Artesunate-Sulphamethoxypyrazine-Pyrimethamine

While we tried to see the relative safety of the RCTs, high statistical heterogeneity precluded the pooled analysis (overall I^2^ = 0.95.8; 9 studies, 16 treatment groups) (Fig. 4).

### Risk of bias assessment

**Fig. 5:**
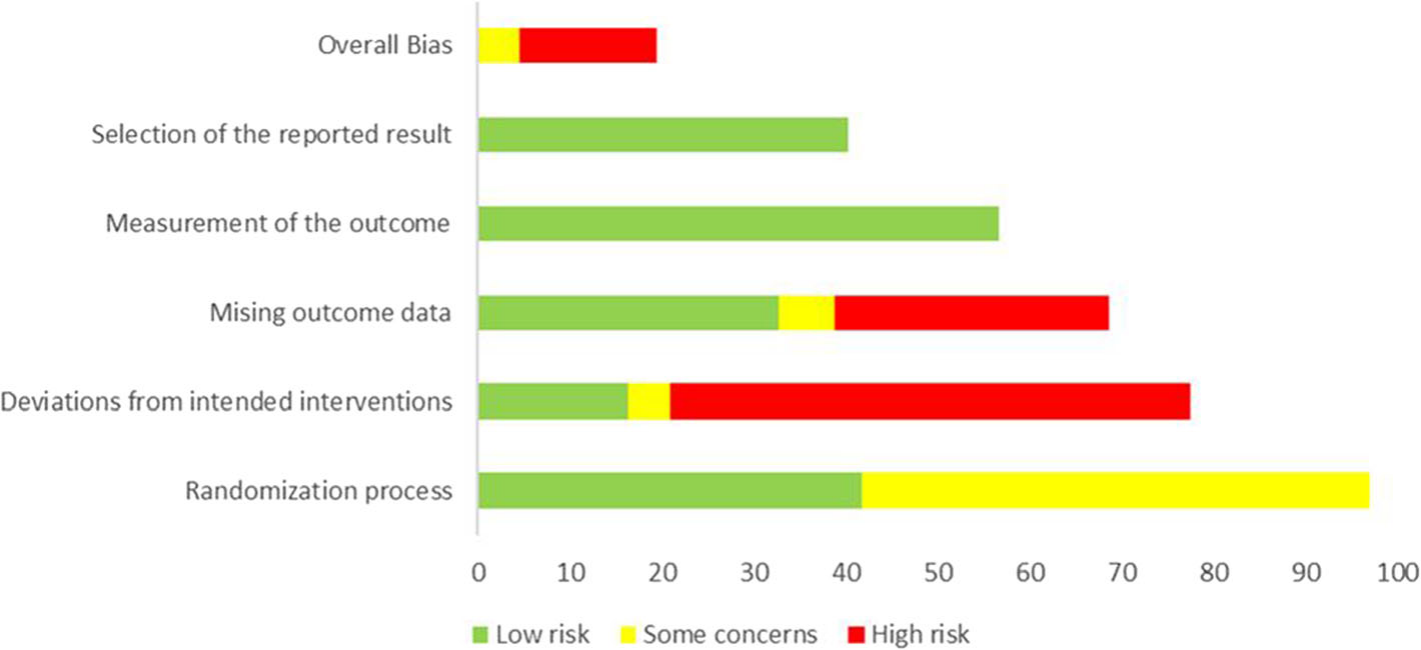
Quality assessment of clinical trial studies included in the systematic review and meta-analysis

The majority of the studies had high bias due to missing outcome data and deviations from intended interventions. Most of the studies were single arm or open label trials and applied no or unclear concealment actions. However, all the RCTs had low bias from selection of the reported results and measurements of the outcome (Fig. 5).

## Discussion

This study attempted to establish the efficacy and safety of ACTs for pediatric uncomplicated malaria management through reviewing and analyzing of the existing body of evidence in the PubMed database until March 06, 2020. Despite the study was planned for falciparum and vivax species, only the falciparum malaria studies were finally analyzed based on the inclusion criteria. Multiple vivax studies were excluded due to presence of other mixed infections. All the studies were assessed based on the WHO protocol, as PCR- corrected day 28 ACPR was provided by all the studies. Based on this, we found that ACTs are still effective and well tolerated for *P. falciparum* malaria management. However, this should be interpreted cautiously as there is a very high heterogeneity among the included studies. This might be because of the inclusion of studies with variable study designs.

The crude overall treatment efficacy of the ACTs was more than 95%, and is acceptable as per the WHO guideline. Except for two treatment arms with AL, all the ACT treatment arms had a treatment failure of less than 10% at day 28 follow-up. Treatment failure is an incapability of administered antimalarial agent to clear malaria parasitaemia or avert recrudescence, irrespective of clinical presentations. WHO recommends a change in the treatment regimen if the treatment failure of an ACT is greater than or equal to 10%. Treatment failure may be attributed to poor patient compliance, incorrect dosage, poor drug quality, and drug interactions or resistance. It is believed that factors contributing for treatment failure are consciously addressed during therapeutic efficacy studies [4].

The two studies with the lower AL efficacy report were a Ghanaian study by Kobbe et al. [29] and a multicenter Angolan study by Plucinski et al. [35]. In the latter, the AL arm in Zaire as opposed to Benguela had low efficacy, however, the age distribution of the included studies vary among the tow AL sites. Only pediatrics < 5 years old. In addition, a similar low AL efficacy was reported in previous study in the area [42]. In both of the studies AL was administered based on the manufacturer’s guideline and no signs of underdosing was reported. Only the first day therapy was directly observed for Kobbe and only the morning doses of the three-day treatment were observed for Plucinski. The Ghanaian study analysis was under powered (65%) due to premature termination of the study secondary to anemia, claimed to be non-drug related.

Although a high heterogeneity excluded the interpretation of efficacy reports for other ACTs, DP showed a very high efficacy in this meta-analysis. The 28-day ACPR cure rate of DP (4 studies) was 99.6% (95% CI: 99.1 to 100%, I^2^ = 0%; 4 studies) and comparable success rate (3 studies) was reported for day 42 (99.6% with 95% CI of 99 to 100%, I^2^ = 0%; 3 studies). Similar to the current study, less than 5% failure rate was reported with DP by Cochrane Reviews [9, 12]. In addition, the WHO database on antimalarial drug efficacy and resistance had showed an overall less than 10% failure rate for DP on day 28 follow-ups. However, a very variable and contradicting very low efficacy reports were included in the WHO database for day 42, particularly from studies in Thailand, Vietnam and Cambodia [43]. This may be due to quality of the studies or settings where the studies were carried. These countries, with high ACT resistance, were under the Greater Mekong Subregion (GMS). Since the initial emergence of partial artemisinin resistance in the region, the GMS have remained the epicentre of antimalarial drug resistance [1]. Despite these, one network meta-analysis in the Asian region including the above-mentioned GMS areas had showed superiority of DP to other ACTs at day 28 with low quality of evidence [44]. Similarly, another network meta-analysis on ACT efficacy for uncomplicated *P. falciparum* malaria in African children and adults showed superiority of DP over other WHO recommended ACTs [45]. By considering the regional resistance disparities, the utility of DP might improve drug compliances as DP is administered once daily without a requirement to take fatty foods. Another interesting point for policy makers is that DP is postulated to decrease malaria incidence in high transmission areas due to its longer prophylactic effect [46].

ACTs are well tolerable than other antimalarial drugs [8, 14]. This systematic review and meta-analysis showed that 9 studies reported ACT- related ADRs (8.3%, 356/4304). The reported drug related ADRs ranged from 1.8% in DP (two studies) to 23.3% in AP (1 study) (Table 5). Similar to other reviews, there was no severe medication-related adverse effect or deaths in all the included studies [5, 7]. The most common adverse effects reported were related to the gastrointestinal system, including, vomiting and diarrhea which resolved spontaneously [5].

The trials included in this review had several limitations. Among these are absence of Kaplan Meier analysis. In addition, most of the included studies were single arm studies and even the RCTs had high risk of bias as described in the result section above.

There were limitations to this review. It is obvious that different search terms will generate different range of articles. Application of filters will also limit the number of studies to be included. In addition, we only searched PubMed. Including studies based on PCR endpoints at day 28 while there could be multiple studies with other measures of efficacy which might be eliminated through the selection criteria from the beginning limits the generalizability of our results. Only the per- protocol analysis in the efficacy review and the intention- to -treat analysis for the safety review were utilized. In addition, the PCR correction techniques were not assessed in this study. Further, the inclusion criteria had also eliminated studies with *P. vivax* species. Only few trials were properly designed and considered high quality to assess treatment success. High level of heterogeneity was also one critical limitation in generating a summary effect. The large effect size of the crude summary effect, however, would offset the observed limitations. Collectively the findings inform that ACTs are still effective for management of pediatric *P. falciparum* malaria. Especially DP is found to be the most efficacies and tolerable choice for falciparum malaria treatment in pediatrics.

## Conclusion

ACTs still demonstrated high treatment success rate and safety for *P. falciparum* although significant heterogeneity precluded generating a summary effect size. In the subgroup analysis, DP showed higher efficacy with no heterogeneity as compared to others. The ACT regimens also showed high tolerability with a low rate of mild and self-limiting ADRs. The high treatment success and tolerability conferred by DP has relevance for policy makers planning the use of ACTs for malaria treatment in the pediatric population.

## Supplementary Information

**Additional file 1: Annex1.** Characteristics of the included studies

## Data Availability

The datasets used and/or analyzed during the current study are presented within the manuscript and/or additional supporting files, and also available from the corresponding author on reasonable request.

## Abbreviations

ACT: Artemisinin-based combination therapies
AL: Artemether–Lumefantrine
CDA: Chlorproguanil-dapsone-artesunate
PCR: Polymerase chain reaction
AP: Artesunate-pyronaridine
DP: Dihydroartemisinin-piperaquine
AQ: Aamodiaquine
AS: Artesunate
CQ: Chloroquine
SP: Sulfadoxine-primethamine
ASAQ: Artesunate-amodiaquine
ACPR: Adequate clinical and parasitological response
PRISMA: Preferred reporting items for systematic review and meta-analysis
PICOS: Participants, interventions, comparisons, outcome measures, study
ASSMP: Artesunate-Sulphamethoxypyrazine-Pyrimethamine
*P. falciparum*: *Plasmodium falciparum*
RCT: Randomized controlled trial
