# Supplementary material for: Efficacy and safety of artemisinin-based combination therapies for the treatment of uncomplicated malaria in pediatrics: a systematic review and meta-analysis
Source: BMC Infect Dis. 2021 Apr 7;21:326. doi: 10.1186/s12879-021-06018-6 (PMC8028735; doi:10.1186/s12879-021-06018-6)
Supplement: Supplementary file 1 — Additional file 1: Annex1. Characteristics of the included studies [file 12879_2021_6018_MOESM1_ESM.docx]

Annex1: Characteristics of the included studies

| **Author, publication year** | **Study period** | **Country** | **Setting** | **Treatments** | **followup (days)** | **enrolled Sample size** | **Missing from PP analysis, n/N(%))** | **Design** | **Age** | **primary outcomes** | **Day 28 PCR corrected ACPR** | **Day 42 PCR corrected ACPR** |
| --- | --- | --- | --- | --- | --- | --- | --- | --- | --- | --- | --- | --- |
|  |  |  |  |  |  |  |  |  |  |  | n/N (P) | n/N (P) |
| Davlantes 2018 | March and July 2017 | Angola (6 sites) | Benguela (stable mesoendemic transmission) | DP, f | upto day 28 & 42 for DP | 100 | 68 (11.2%) of 608 | multicentre, 6 arm, semi-comparative CT | <12 years on two sites and <5 years on the rest | ACPR, failure to adequately clear parasitaemia, or recurrent parasitaemia | 85/85 (100) | 80/80 (100.0) |
|  |  |  |  | ASAQ, f |  | 105 |  |  |  |  | 90/90 (100) |  |
|  |  |  | Zaire (stable mesoendemic transmission) | AL, f |  | 100 |  |  |  |  | 87/91 (95.5) |  |
|  |  |  |  | ASAQ, f |  | 98 |  |  |  |  | 74/79 (93) |  |
|  |  |  | LundaSul-hyperendemic transmission) | AL, f |  | 105 |  |  |  |  | 82/85 (96.4) |  |
|  |  |  |  | DP, f |  | 100 |  |  |  |  | 89/89 (100) | 89/89 (100) |
| Nhama 2014 | cohort 1 June 2011, and cohort 2 august 2012 | Mozambique (5 sites) | 4 hospitals and 1 HC, perennial, non-pick transmission periods selected | AL, f | 1, 2, 3, 7, 14, 21 and 28 | 439 | 94/700 ( 66/439 AL and 28/261 ASAQ) | multisite, open-label, non-RCT, two-cohort, CT | 6 to 59 months | PCR-corrected ETF, LCF, LPF and ACPR at day 28 | 335/349 (96) |  |
|  |  |  |  | ASAQ, f |  | 261 |  |  |  |  | 232/233 (99.6) |  |
| Ojurongbe 2013 | February 2010 and September 2011 | Nigerian | University hospital, throughout the year | AL, f | 3, 7, 14, 21 & 28. | 89 | 22/182, 12/101 form Coartem& 10/81 Larima arm. | RCT (open label, comparative) | 6 and 144 months | ACPR | 89/89 (100) |  |
|  |  |  |  | ASAQ, f |  | 71 |  |  |  |  | 71/71 (100) |  |
| Plucinski 2017 | January to June 2015 | Angola (Urban, public health facilities) | Benguela (stable mesoendemic transmission) | ASAQ | 1,2,3, 7,14,21, 28 for all and 42 for DP | 101 | 119/586 (50/179 from ASAQ, 27/205 AL & 42/202 from DP) | non-randomized, six-arm non comparative trial | 6 to 59 months in Zaire and LundaSul, and 6 months to 12 years in Benguela. | ACPR | 66/66 (100) |  |
|  |  |  |  | AL, f |  | 101 |  |  |  |  | 76/78 (97.4) |  |
|  |  |  | Zaire (stable mesoendemic transmission) | AL, f |  | 104 |  |  |  |  | 70/81 (86.4) |  |
|  |  |  |  | DP, f |  | 102 |  |  |  |  | 80/81 (98.8) | 98.5 |
|  |  |  | LundaSul-hyperendemic transmission) | DP, f |  | 100 |  |  |  |  | 76/76 (100) | 100 |
|  |  |  |  | ASAQ |  | 78 |  |  |  |  | 56/56 (100) |  |
| Premji 2009 | June 2006 and August 2007 | (Burkina Faso, Ghana, Kenya, Nigeria, Tanzania) | medical centers at 11 sites in five Africa | CDA | 1, 2, 3, 7, 14, 28 & 42. | 914 | 61/1732 lost, (44/914 CDA & 17/458 in AL) | RCT, Double-Blind, Phase III Trial | 1to 15 years | parasitological cure (PCR-corrected) at Day 28, | 708/765 (92.5) | 697/771 |
|  |  |  |  | AL |  | 458 |  |  |  |  | 360/381 (94.5) | 358/384 |
| Ramharter 2008 | June to December 2006 | Gabon | Hospital, transmitted perennially | AP 9:3-PA mg/kg tabs | Until day 42. | 15 | 7/59 | open-label sequential dose escalation | 2 to 14 years | tolerability, safety, and pharmacokinetics | 13/13 (100) | 8/9 (89) |
|  |  |  |  | AP 9:3-mg/kg tabs |  | 15 |  |  |  |  | 14/14 (100) | 13/14 (100) |
| Sawa 2013 | April to June 2009 | Kenya | moderate transmission | AL, f | 1, 2, 3, 7, 14, 28, & 42. | 153 | 14/198 (6/153 form AL & 8/145 form DP) | RCT | 6months to 10 years | efficacy of AL and DP | 137/139 (98.6) |  |
|  |  |  |  | DP, f |  | 145 |  |  |  |  | 137/137 (100) |  |
| Shayo 2014 | May and August 2013 | Tanzania | Health Center. Moderate to high transmission | AL, f (< 5 years) | 1, 2, 3, 7, 14, 21, & 28. | 56 | 9/88 | Prospective, open-label,non-randomized single-arm trial | 6 months to 10 years | parasitological cure on day 28 | 40/40 (100) |  |
|  |  |  |  | AL, f (5 and above) |  | 32 |  |  |  |  | 21/21 (100) |  |
| Sirima 2009 | October 2004 to February 2006 | Burkina Faso | two medical centers over two malaria seasons | ASAQ, f | 1, 2, 3, 7, 14, 21 & 28 | 375 | 124/750 (65/375 in AS/AG & 59/375 in AS-AQ) | RCT | 6 to 59 months | PCR-corrected ACPR based on the PP | 315/342 (92.1) |  |
|  |  |  |  | AS+AQ, l |  | 375 |  |  |  |  | 313/340 (92.1) |  |
| Thwing 2009 | 14th May to 24th August 2007 | Kenya | District Hospital, during the rainy season | ASAQ, l | 1, 2, 3, 7, 14, 21 & 28 | 110 | 7/110 | Single arm efficacy trial | 6 to 59 months |  | 92/102 (90.2) |  |
| Ndounga 2015 | April 2010 to March 2011 | Republic of Congo | suburban, district hospital, intense and perennial transmission | ASAQ, f | 1, 2, 3, 7, 14, 21 & 28 | 129 | 38/282 lost (23/129 in ASAQ and 15/133 in AL) | Two arm randomized trial | under 10 years | PCR-unadjusted and PCRadjusted cure rates on day 28 | 96/99 (97) |  |
|  |  |  |  | AL, f |  | 133 |  |  |  |  | 106/110 (96.4) |  |
| Mens 2008 | April-July 2007 | Kenya | health center, high transmission season | DP, f | 1, 2, 3, 7, 14, & 28 | 73 | 12/146 lost | Randomized controlled trial, PG | 6 months-12 years |  | 67/67 (100) |  |
|  |  |  |  | AL, f |  | 73 |  |  |  |  | 66/66 (100) |  |
| Menard 2007 | February and June 2006 | Madagascar | primary health centers, low & predominantly seasonal transmission | CQ | 1, 2, 3, 7, 14, 21, 28, | 42 | 22/287 lost, (4/73 from AQSP and 7/83 from AQAS) | RCT | 6 months to 15 years | day-14 and day-28 risks of parasitological failure | 20/36 (55.6) |  |
|  |  |  |  | AQ |  | 39 |  |  |  |  | 35/36 (97.2) |  |
|  |  |  |  | SP |  | 40 |  |  |  |  | 37/38 (97.4) |  |
|  |  |  |  | AQ + SP |  | 83 |  |  |  |  | 76/79 (96.2) |  |
|  |  |  |  | AS+AQ |  | 83 |  |  |  |  | 70/76 (92.1) |  |
| Kobbe 2008 | October 2006 and September 2007 | Ghanaian | two district hospitals, holoendemic and perennial with seasonal peaks | AL, f | 3, 7, 14, 28 | 120 | 47/246 (20/123 in AL and 27/123 in ASAQ) | RCT, open-label trial, | 6 to 59 months | 28-day risks of recurrent symptoms &parasitaemia (ETF, LCF, LPF), unadjusted & adjusted) | 91/103 (88.3) |  |
|  |  |  |  | ASAQ, coblistered |  | 117 |  |  |  |  | 88/96 (91.7) |  |
| Kabanywanyi 2007 | 2004 (Ipinda: Jan and June and Milmba: March and Oct at Ipinda and Mlimba) | Tanzania | health facilities, perennial with seasonal picks | AL, f (Ipinda site) | 1, 2, 3,7,14, 21 & 28 | 99 | 18/175 (7/99 (7%) in AL & 11/76 (14.5%) in ASAQ) | non randomized trial | 6 to 59 months |  | 86/86 (100) |  |
|  |  |  |  | ASAQ, l (Mlimba site) |  | 76 |  |  |  |  | 45/48 (93.8) |  |
| Dorkenoo 2012 | 2005, 2007, 2009 (Aug to Nov at Lome&Octo&Decr for other sites) | Togo (5 sites) | 4 hospitals, high transmission season | ASAQ, l | up to day 28. | 651 | 37/1296 (19/651 in ASAQ , 18/645 in AL) | multi-arm (5) trial | 6 to 59 months | 28-day PCR corrected cure rate, No ITT, KM for TF | 580/600 (96.7) |  |
|  |  |  |  | AL, f |  | 645 |  |  |  |  | 538/538 (100) |  |
| Dorkenoo 2016 | Oct to Dec 2012 in Niamtougou&Sokod& June to August 2013 in Lome. | Togo (3 sites) | Hospitals, high transmission season selected | ASAQ, f | 1, 2, 3, 7, 14, 21 & 28. | 262 | 19/523 (3 from ASAQ & 16 from AL) | multi-arm prospective study , PG | 6 to 59 months | 28-day PCR corrected ACPR | 249/252 (98.8) |  |
|  |  |  |  | AL, f |  | 261 |  |  |  |  | 220/224 (98.2) |  |
| Ayede 2010 | Not reported | Nigeria | Urban, university and child’s hospital, endemic or seasonal transmission | ASSMP, f | 1, 2, 3,7, 14, 21 & 28 | 250 | 33 (6.6%), (21 in AS + SMP and 12 in AS+ AQ) | randomized, controlled, open-label trial, PG | 1 to 13 years | PCR-corrected ACPRon day 28 | 219/229 (95.6) |  |
|  |  |  |  | ASAQ,f |  | 250 |  |  |  |  | 233/238 (97.9) |  |
| Abuaku 2019 Abuaku 2019 | not reported | Ghana | 9 hospitals, 1 polyclinic, is perennial transmission (intense & not intense) | AL, f | 1, 2, 3, 7, 14, 21, & 28. | 472 | 26/472 | one arm, prospective, evaluation | 6months to 9 years | Prevalence of day 3 parasitaemia, ACPR on day 28. | 405/422 (96) |  |
|  |  |  |  | ASAQ, f |  | 492 | 19/492 |  |  |  | 466/470 (99.2) |  |

*Abbreviations:- ACPR: adequate clinical and parasitological response, ACT: artimisinin combination therapy, PCR: polymerase chain reaction, RCT: randomized controlled trial, f: fixed-dose combination, l: loose combination, AL: Artemether–Lumefantrine, ASAQ: Artesunate–Amodiaquine, DP: dihydroartemisinin-piperaquine, CDA:* Chlorproguanil-dapsone-artesunate,*AP: artesunate-pyronaridine, ASSMP: Artesunate-Sulphamethoxypyrazine-Pyrimethamine, CQ: Chloroquine, AQ: amodiaquine, SP: sulfadoxine-primethamine*
